# Recent Advances on Rare Earth Upconversion Nanomaterials for Combined Tumor Near-Infrared Photoimmunotherapy

**DOI:** 10.3389/fchem.2020.596658

**Published:** 2020-11-06

**Authors:** Weilin Chen, Yulin Xie, Man Wang, Chunxia Li

**Affiliations:** Institute of Frontier and Interdisciplinarity Science, Institute of Molecular Sciences and Engineering, Shandong University, Qingdao, China

**Keywords:** upconversion nanoparticles, near infrared, immunotherapy, phototherapy, combined therapy

## Abstract

Cancer has been threatening the safety of human life. In order to treat cancer, many methods have been developed to treat tumor, such as traditional therapies like surgery, chemotherapy, radiotherapy, as well as new strategies like photodynamic therapy, photothermal therapy, sonodynamic therapy, and other emerging therapies. Although there are so many ways to treat tumors, these methods all face the dilemma that they are incapable to cope with metastasis and recurrence of tumors. The emergence of immunotherapy has given the hope to conquer the challenge. Immunotherapy is to use the body's own immune system to stimulate and maintain a systemic immune response to form immunological memory, resist the metastasis and recurrence of tumors. At the same time, immunotherapy can combine with other treatments to exhibit excellent antitumor effects. Upconversion nanoparticles (UCNPs) can convert near-infrared (NIR) light into ultraviolet and visible light, thus have good performance in bioimaging and NIR triggered phototherapy. In this review paper, we summarize the design, fabrication, and application of UCNPs-based NIR photoimmunotherapy for combined cancer treatment, as well as put forward the prospect of future development.

## Introduction

Malignant tumor is one of the major diseases that seriously threaten human health. Because of the metastasis and recurrence of tumor, it is still a great challenge to eliminate cancer completely. The outcome of the persisting declination in cancer death rates since 1991 was an over drop of 27% (Siegel et al., 2019). In the next 20 years, new cancer cases and deaths are expected to increase to 22 million and 13 million, respectively. Thus, great efforts have been devoted to developing new approaches for the diagnosis and treatment of tumors. Modern oncology researches show that tumor is not a simple local disease, but a local manifestation of systemic disease. It is difficult to remove all tumor cells in the body by the existing tumor treatment methods. The conventional strategies including chemotherapy and radiotherapy often lead to the tolerance of tumor cells to the treatment. Consequently, the recurrence and metastasis of tumors are inevitable after treatment.

Immunotherapy has recently become an effective cancer treatment strategy. In 2018 the Nobel Prize in Physiology or Medicine was awarded to the field of cancer for the discovery of cancer therapy by inhibition of negative immune regulation (Hokland et al., 2018). The purpose of immunotherapy is to use the body's own immune system to stimulate and activate systemic immune response, specifically eliminate minimal residual disease, resulting in inhibiting tumor growth and breaking the immune tolerance (Shekarian et al., 2015; Hu et al., 2019b; Chang et al., 2020). In other ways, tumor immunotherapy is to overcome the mechanism of tumor immune escape, so as to re-awake immune cells to clear cancer cells. Because of its slight side effects and obvious therapeutic effect, immunotherapy has gradually become the development direction of tumor treatment in the future, which is regarded as the fourth major tumor treatment technology after surgery, radiotherapy and chemotherapy (Yang et al., 2016).

Although immunotherapy has made considerable progress, the effect of immunotherapy is challenged by the immune escape mechanism of tumor, which brings the establishment of immunosuppressive microenvironment through the loss of antigenicity and immunogenicity (Beatty and Gladney, 2015). Moreover, immunotherapy has a wide range of targets, and side effects are inevitable. For example, immune checkpoint blockade raises the immune system to a high level while also attacking normal cells (Sang et al., 2019). Therefore, immunotherapy is generally used as an adjuvant therapy in combination with other traditional therapies to improve the comprehensive therapeutic effect and prevent recurrence and metastasis of the tumor. In this case, the integrated cancer therapy will be the new development direction of immunotherapy.

Phototherapy including photodynamic therapy (PDT) and photothermal therapy (PTT) has always been a research hotspot (Gai et al., 2018). PTT could employ PTT agents to convert light energy into hyperthermia to precisely “cook” cancer cells (Zhang et al., 2016; Ding et al., 2017; Gao et al., 2017; Wang et al., 2017). The three elements of PDT are photosensitizer (PS), oxygen, and light. The principle of PDT is to irradiate the tumor site with a specific wavelength to activate the photosensitive drugs that selectively gather in the tumor tissue, and induce the production of cytotoxic reactive oxygen species (ROS) by luminescent chemical reaction, and induce tumor death (Henderson and Dougherty, 1992; Sharman et al., 1999; Juarranz et al., 2008; Lan et al., 2019). The process of phototherapy can induce the antitumor immune response by inducing apoptosis or necrosis of cancer cells to induce immunogenic cell death (ICD) and release tumor associated antigen (TAA). This “*in situ* vaccine” strategy can stimulate “cold” tumor microenvironment (TME) to become “hot” immunogenic TME (Wang et al., 2018b, 2020a; Duan et al., 2019; Deng et al., 2020). Thus, the marrying of immunotherapy and phototherapy provides new opportunities for the treatment of tumors with high efficacy (Chang et al., 2019; Kobayashi and Choyke, 2019; Ma et al., 2019).

However, the traditional PDT PSs are usually excited by ultraviolet (UV) or visible light. In view of the challenges of these wavelengths such as lower penetration depth of biological tissue, higher background luminescence interference, and stronger light damage to normal tissues, the application of PDT in biomedical field is limited to some extent (Wang et al., 2004; Quail and Joyce, 2013; Zhou et al., 2016). In contrast, NIR light can address these problems and be used as a good minimally invasive manner and external stimulation style for light triggered delivery of various therapeutic agents for the diagnosis and treatment of tumors (Gai et al., 2014; Zheng et al., 2015; Guryev et al., 2018; Wang et al., 2018a, 2020b; Chen et al., 2019). Rare earth UCNPs can effectively convert NIR light into UV, visible light even NIR light under the excitation of 980/808 nm NIR as excitation light source (Sun et al., 2014; Zhong et al., 2014; Dong et al., 2015; Liu et al., 2017; Gu et al., 2019; Rabie et al., 2019). In light of the special advantages of NIR light, UCNPs can serve as optical probe for the tracking of *in vivo* treatment, but also as energy transducers to indirectly activate PS to generate ROS for PDT (Liu et al., 2014; Yang et al., 2015; Hou et al., 2016, 2019; Xu et al., 2018a; Feng et al., 2019; Tang et al., 2019). In addition, UCNPs make it possible to modulate photosensitive switches by NIR light due to their unique upconversion luminescence (UCL) ability (Zou et al., 2012). The modulation of photosensitive switches with UCNPs could overcome the penetration depth and photoactivation efficiency challenged by UV and visible light. Thus, NIR light has become an attractive tool for precisely regulating the time and space of chemical and biological activities (Kowalik and Chen, 2017; Ankenbruck et al., 2018).

Although PDT could generate certain levels of immune responses, the immune effects from PDT alone are not enough for inhibiting the remaining tumor growth (Castano et al., 2006). Thus, the combined PDT and immunotherapy would achieve an enhanced cancer treatment outcome (Xu et al., 2020). This is also consistent with the idea of multi-synergistic therapy, that is, the use of two toxic drugs will lead to superadditive effect, and the total effect of drugs (R_ab_) is greater than the arithmetic sum of constituent toxic effects (R_a_, R_b_), making R_ab_ > R_a_ + R_b_ (Gordon Steel and Peckham, 1979; Seiwert et al., 2007). Although there exist pharmacokinetic inconsistencies, long-term studies on combination therapy have confirmed its effect (Wang et al., 2019b). In this review, focusing on the combination between UCNPs mediated phototherapy and immunotherapy, the current advancements and latest breakthroughs have been summarized. Furthermore, the major challenges in the combinatorial strategies and the future prospects are also highlighted. The composition of UCNPs combined with immunotherapy was summarized in Table 1. Though the combination of phototherapy and immunotherapy emerged early, the use of UCNPs with immunotherapy is just springing up. In particular, we hope that this review could shed light on the development of the combination between UCNPs mediated phototherapy and immunotherapy for high performance tumor therapy.

**Table 1:** Summary of UCNPs combined with immunotherapy.

**Nanocarrier**	**UCNPs**	**Immunotherapy agents**	**References**
UCNP-PEG-PEI	NaY/GdF_4_ (Y:Gd:Yb:Er = 58%:20%:20%:2%)	OVA	Xiang et al., 2015
Monodispersed macroporous mesoporous silica-coated upconversion nanoparticles (UCMSs)	β-NaYF_4_:20%Yb,2%Er	OVA/tumor cell fragment (TF)	Ding et al., 2018
SA-PEG-TK-PLGA (SPTP)	β-NaYF_4_:20%Yb,2%Er	DOX and RB	Jin et al., 2020
UCNPs	NaYF_4_:20%Yb,2%Er	R837 and CTLA-4 immune checkpoint inhibitors	Xu et al., 2017a
UCNP@SiO_2_	NaYF_4_:Yb,Er	Anti-CTLA-4	Lin et al., 2020
UCNPs	NaYF_4_:48%Yb/2%Er@NaYF_4_:30%Nd Core/Shell Nanoparticles	Anti-CTLA-4	Wang et al., 2019a
PDA@UCNP-PEG	NaGdF4:Yb/Er	α-PD-1	Yan et al., 2019
AIE luminogen (AIEgen)–coupled UCNPs (AUNPs)	NaYF_4_:Yb/Tm@NaYF_4_	α-PD-1	Mao et al., 2020
UCNPs@porphyrin MOFs (UCSs)	NaGdF_4_:Yb,Er@NaGdF_4_	α-PD-L1	Shao et al., 2020
UCNPs	NaGdF_4_:Yb,Er@NaYF_4_@NaYF_4_:Yb,Tm@NaYbF_4_:Nd@NaYF_4_	α-PD-L1	Di et al., 2020
UCNPs	NaGdF_4_:70%Yb,1%Tm@NaGdF_4_ core-shell UCNPs	CpG ODNs	Chu et al., 2019
UCNPs@mesoporous silica	NaYF_4_:Er/Yb	CCL21	Lee et al., 2013
UCNs-MnO_2_	NaYF_4_:Yb/Tm/Nd (30/0.5/1%)@NaYF_4_:Nd (20%)	HA	Ai et al., 2018
Bi doped mesoporous upconversion nanophosphor (UCNP)	Na_0.2_Bi_0.8_O_0.35_F_1.91_:20%Yb,2%Er	DOX and X-ray	Qin et al., 2020

## Classification of Immunotherapy

Concomitant with the development of the immunotherapy, it has five kinds of representative treatment modalities.

First of all, antibody therapy has gradually become one of the standard regimens for cancer treatment, which mainly includes tumor targeted antibody drug therapy and immunomodulatory antibody therapy based on the different ways of activating the antitumor immune response. The mechanism of the former is to target the cellular growth factor receptors and cell surface antigens, etc. At present, many tumor targeting antibodies have been approved by Food and Drug Administration (FDA). For example, Avastin can effectively block the binding of vascular endothelial growth factor and receptor, inhibit the formation of tumor blood vessels, and achieve antitumor effect (Sullivan and Brekken, 2010). In contrast, the targets of immunomodulatory antibodies are immune cells rather than tumor cells, which can augment the antitumor immune response of the body by blocking the immunosuppressive pathway or directly playing the role of immune stimulation. Among various immunomodulatory antibodies, antibody blockers for inhibitory receptors mainly act on the immune checkpoints, which have shown encouraging therapeutic effects (Pardoll, 2012). Cytotoxic T-lymphocyte-associated protein 4 (CTLA-4) and programmed death 1 (PD-1) are two kinds of crucial inhibitory regulatory receptor that are overexpressed on the activated T cells (Dariavach et al., 1988; Francisco et al., 2010). Activation of T lymphocytes requires two signals: the major histocompatibility complex (MHC) peptide signal and costimulatory molecule signal. Costimulatory molecules mainly include positive costimulatory CD27, CD28, and CD137 pathways as well as negative costimulatory CTLA-4 and PD-1/programmed death 1 ligand (PD-L1) pathways to prevent T cells from being over stimulated. This inhibition pathway can be hijacked by tumor to fight the immune system. Therefore, the use of positive costimulatory factor agonists or negative costimulatory factor antagonists can improve the immune killing effect of T cells on tumor. Ipilimumab is the first checkpoint antibody was approved by the FDA in 2011 for treatment of melanoma (Cameron et al., 2011). Ipilimumab can block the action of CTLA-4 molecules on the surface of activated T cells, so that T cells can maintain sustained antitumor activity. At present, antibody therapy has become a mature and widely used cancer treatment method.

The second kind of immunotherapy therapy is biological response modulators (BRMs), also known as immunomodulators or immunopotentiators, which involves those components that can improve the activity level of the immune system, thus enhancing the effect of immunotherapy. BRMs include a variety of cytokines, toll like receptor (TLR) signaling and non-coding RNAs. Cytokine is one of the typical non-specific BRMs. Some cytokines have been approved by FDA for cancer treatment. For example, Interleukin 2 (IL-2) is a kind of representative cytokines, which could regulate the survival, proliferation, and differentiation of T cell and natural killer (NK) cell, which could be put to use in the treatment of malignant melanoma and renal cell carcinoma (Dranoff, 2004). In addition to cytokines, TLR signaling is also an important biological response regulator, which can be activated by pathogen-associated molecular patterns or similar agonists, and then activate downstream signaling to induce immune response to eliminate pathogens (Kawasaki and Kawai, 2014). TLR agonists have been used in different cancers. For example, in breast cancer, colorectal cancer, and lung cancer, TLR3 agonists could induce tumor cell apoptosis through caspase (Salaun et al., 2006). Non-coding RNA plays a regulatory role in the development, differentiation, and activation of infiltrating leukocytes in tumor tissue (Desgranges et al., 2019). Non-coding RNA based therapy can regulate the TME to improve the effect of tumor therapy. At present, MRX34, the first miRNA-based tumor therapeutic agent, has entered phase I clinical trials (Bouchie, 2013).

Tumor vaccine is also one of the methods of tumor immunotherapy, which can be divided into preventive tumor vaccine and therapeutic tumor vaccine. Among them, the therapeutic vaccine for tumor antigen has been developed rapidly. The common tumor vaccines include protein/peptide vaccines, cell vaccines, DNA vaccines, and so on (Hu et al., 2018b). Protein/peptide vaccines are the most common kinds, which could be recognized by antigen-presenting cells (APCs) and activate T cells to kill cancer cells expressing antigen proteins. Cell vaccines could activate the immune response by injecting inactivated tumor cells or dendritic cells (DCs) expressing tumor antigens. DNA vaccines are prepared by injecting DNA into cells by gene technology, which makes cells produce antigen directly and cause immune protection. After years of development, many therapeutic tumor vaccines have been put into clinical practice and even to the market. For example, provenge vaccine and M-vax vaccine have been approved for marketing and have achieved good results in clinical applications (Berd, 2004; Anassi and Ndefo, 2011).

Adoptive cell therapy is a new type of immunotherapy, which depends on tumor specific T cells. The T cells cultured and activated *in vitro* can be reinfused into the body to kill the tumor (Kalos and June, 2013). Previously, adoptive cell therapy also included the use of NK cells, cytokine induced killer cells, and tumor infiltrating lymphocytes, and so on. The earliest adoptive cell therapy strategy employed lymphokine-activated killer cells, which use IL-2 to stimulate and activate peripheral blood mononuclear cells *in vitro*. This method was approved by FDA as early as 1984 (Mazumder and Rosenberg, 1984). With the continuous development and innovation of cell engineering technology, T cells improved by cell engineering have gradually become the focus of adoptive cell therapy. T-cell receptor engineered T-Cell (TCR-T) can improve the recognition and clearance of cancer cells with the antigen which express the designed T cell receptor. Chimeric antibody receptor engineered T Cell (CAR-T) could recognize a specific protein expressed on the surface of tumor cells and lead to rapid activation and tumor cell killing (Ikeda, 2016; June et al., 2018). Both of these cell-engineered modified T cells have achieved good results in clinical trials (Robbins et al., 2011; Maude et al., 2014).

Oncolytic immunotherapy is a newly developing kind of tumor immunotherapy. Significant success has been achieved through the use of oncolytic immunotherapy, which could cause virus-induced tumor cell death and release of relevant antigens. Oncolytic immunotherapy could induce of long-term immune response formation, especially in combination with other immunotherapies (Beug et al., 2014; Workenhe et al., 2014). At present, there are 67 studies about oncolytic virus on the official website of clinical trials (clinicaltrials.gov), indicating that oncolytic virus has potential as one of the treatment methods for cancer.

## Fabrication and Design of UCNPs-Based Photoimmunotherapy Paltform

In this review, all of the UCNPs are synthesized by thermal decomposition method or urea coprecipitation method. In general, the keys to a successful synthesis are carefully choosing precursors, tuning of the coordinating behavior of the solvents using coordinating and non-coordinating surfactants, and maintaining balance between nucleation and growth stages (Mai et al., 2006). Thermal decomposition method is to obtain UCNPs by cracking rare earth salt precursors in high temperature and shielding gas. While urea coprecipitation method could obtain crystalline UCNPs directly by coprecipitation nanoparticles within fluid solvent. These issues requiring attention could be controlled by both of them to obtain nanocrystalline materials with uniform, controllable, and single dispersion.

The design of the UCNP-based therapeutic nanoplatform should give full play to the unique characteristics of UCNPs, and combine multiple functional modules to achieve treatment and diagnosis. How to realize the effective connection of functional modules and maximize the role of functional groups is a challenge (Yang et al., 2015). First of all, the synthesized UCNPs should be well-shaped, monodisperse and homogeneous. High quality UCNPs are essential for the synthesis of high-efficiency therapeutic platform. Secondly, as kind of nanocomposites, UCNP-based therapeutic nanoplatform needs to connect different substances, which could be achieved through a variety of ways, such as electrostatic force, hydrophobic force, and chemical bond formation. Meanwhile, UCNPs need to be modified according to the properties of immune functional modules, so as to realize the construction of photoimmunotherapy nanoplatform. Thirdly, the key to reasonable design is to perform each function module. UCNPs can be used as carriers to carry photothermal agents, chemotherapy drugs, immune adjuvants, etc., and can also be used as activators to convert near-infrared light into ultraviolet and visible light to activate PS. Finally, UCNPs-based nanoplatforms need good biocompatibility and targeting to achieve good results.

## The Corpration of UCNPs and Immunotherapy

**Figure 1:**
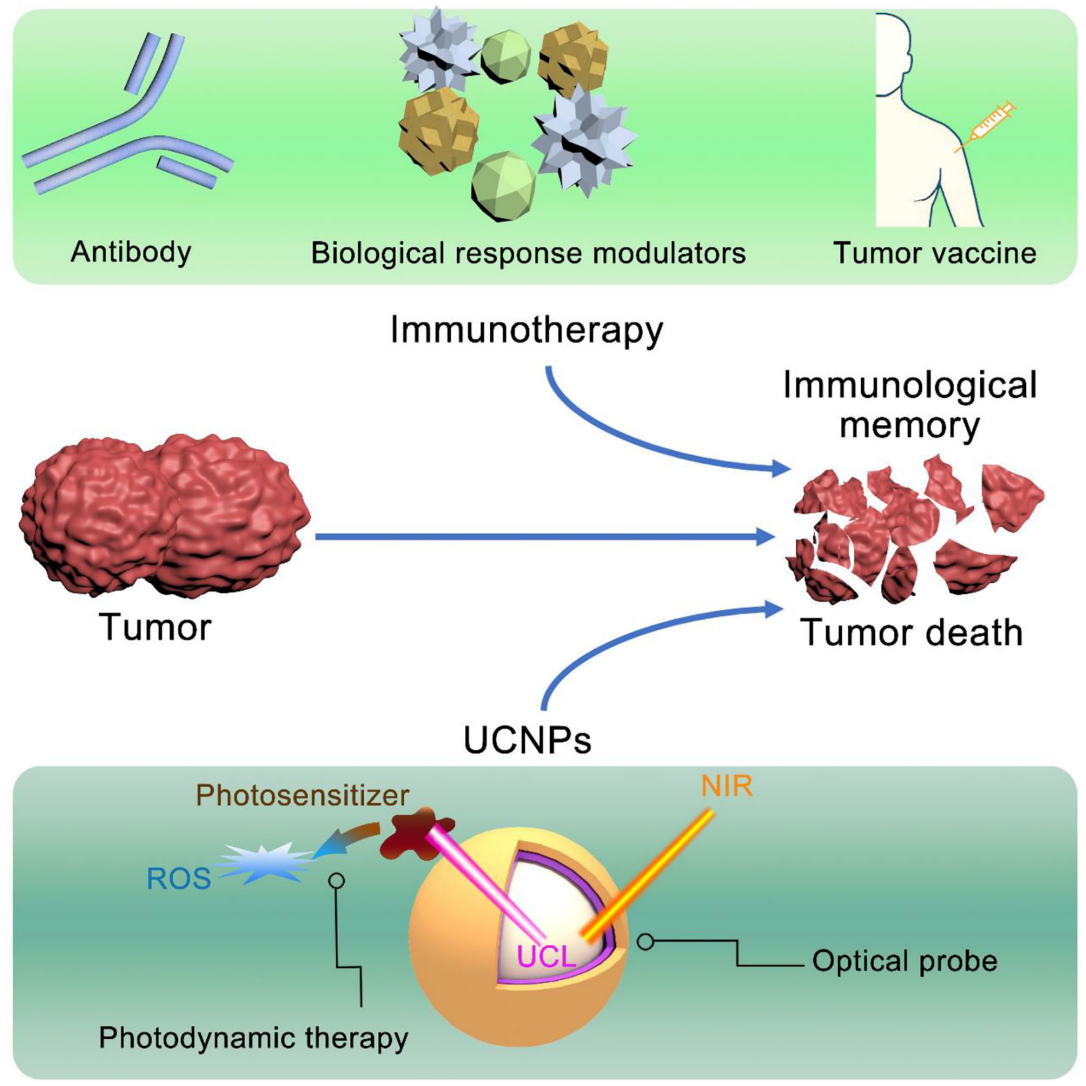
The combination of UCNPs and immunotherapy.

In this section, we summarized the design strategy of UCNPs-based nanomaterials in combination with immunotherapy for synergistic therapy of tumors, as indicated in the Figure 1.

### UCNPs With Vaccine and Vaccine-Like Therapy

**Figure 2:**
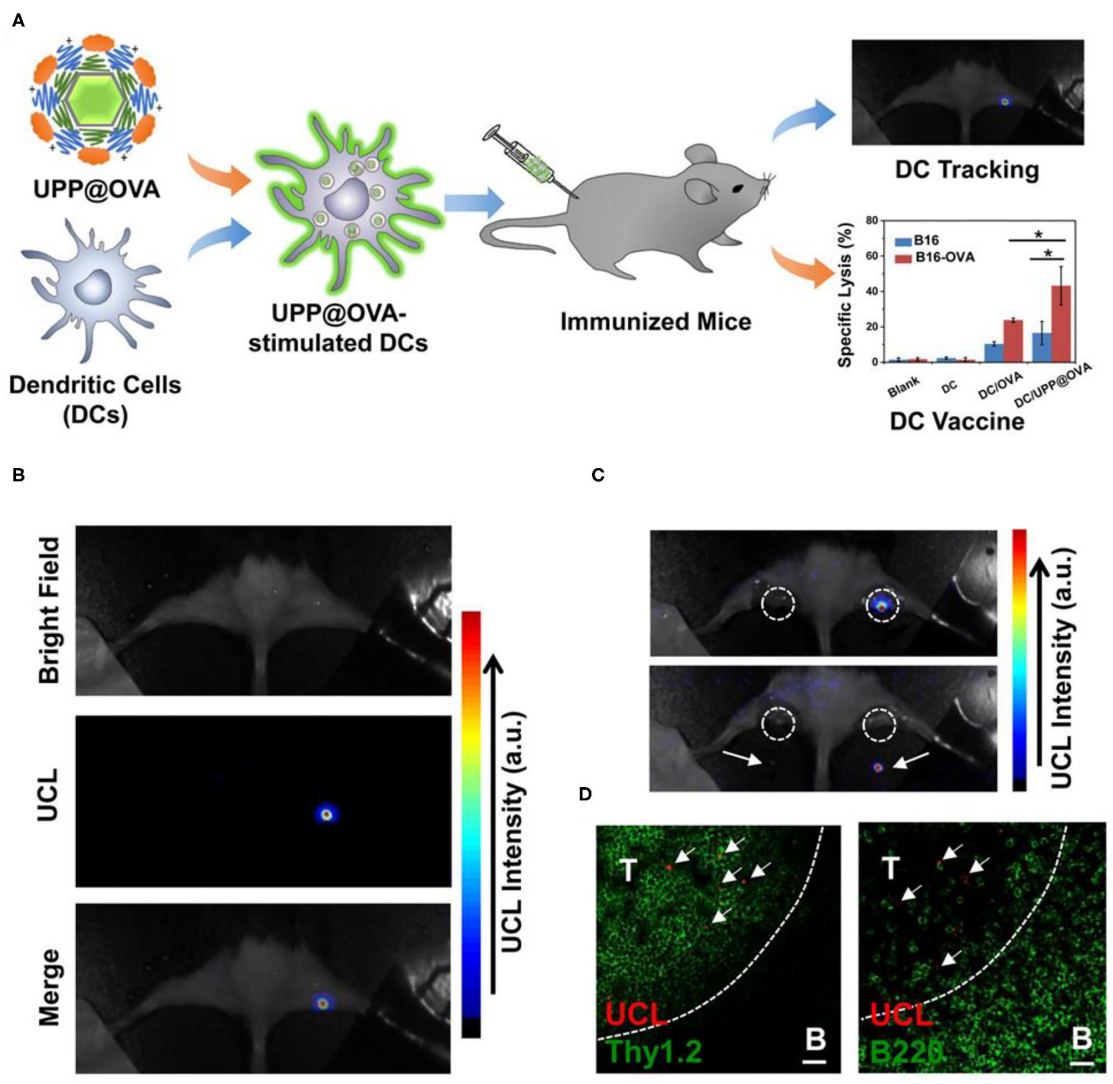
**(A)** Schematic illustration of antigen-loaded UCNPs for DC stimulation, tracking, and vaccination in DC-based immunotherapy. **(B)** Labeled DCs were injected into the right footpad of a mouse; 48 h after injection, strong UCL signals from the draining lymph node were seen under *in vivo* UCL imaging. **(C)** *Ex vivo* imaging of popliteal lymph nodes (white circles) before (top) and after (bottom) being dissected from the mouse. **(D)** Immunofluorescence images of the lymph nodes dissected from the mouse injected with UPP@OVA-labeled DCs. T, T-cell zone (Thy1.2þ); B, B-cell zone (B220þ). Scale bar = 20 μm. Arrows point to UCL signals from labeled DCs. Copyright 2015, American Chemical Society (Xiang et al., 2015). *P < 0.05.

DCs are powerful antigen presenting cells *in vivo*. With surface expressing MHC class I/II molecules and T-cells activation costimulatory molecules, etc., DCs have antigen uptake, processing, and presentation functions (Kapsenberg, 2003; Steinman, 2012). DCs can be divided into immature DCs and mature DCs according to their functions. When antigen was presented to DCs, immature DCs differentiated into mature DCs. Then, DCs loaded with antigen peptide migrated to lymph nodes and activated T cells (Banchereau et al., 2000; Randolph et al., 2005). The migration process is the key process to DC vaccine. However, traditional imaging methods cannot achieve real-time imaging of DC migration. Xiang et al. used UCNPs to load antigens to label and stimulate DCs (Xiang et al., 2015). The location of DC vaccine can be traced through UCL imaging *in vivo* during DC immunotherapy. Firstly, they modified UCNPs (NaY/GdF_4_:Yb,Er) with polyethylene glycol (PEG) and polyethyleneimine (PEI) to prepare UCNP-PEG-PEI (UPP). Then, the antigen model OVA was loaded onto UPP (UPP@OVA) by electrostatic interaction (Figure 2A). Mature DCs were injected into tumor-bearing mice, and the location of DCs could be traced by UCL imaging *in vivo*, revealing the homing process of DCs from peripheral tissues to draining lymph nodes (Figures 2B,C). Compared with OVA-treated DC vaccine, the UPP@OVA treated DC vaccine elicited strong antigen-specific immune responses (Figure 2D), such as enhanced T cell proliferation, IFN-gamma (IFN-γ) production, and cytotoxic T lymphocyte (CTL)-mediated responses. This work is the first to achieve highly sensitive *in vivo* DC tracing and prepare DC vaccines with strong immune function using antigen-loaded UCNPs, which is of great significance in the development of traceable DC immunotherapy.

**Figure 3:**
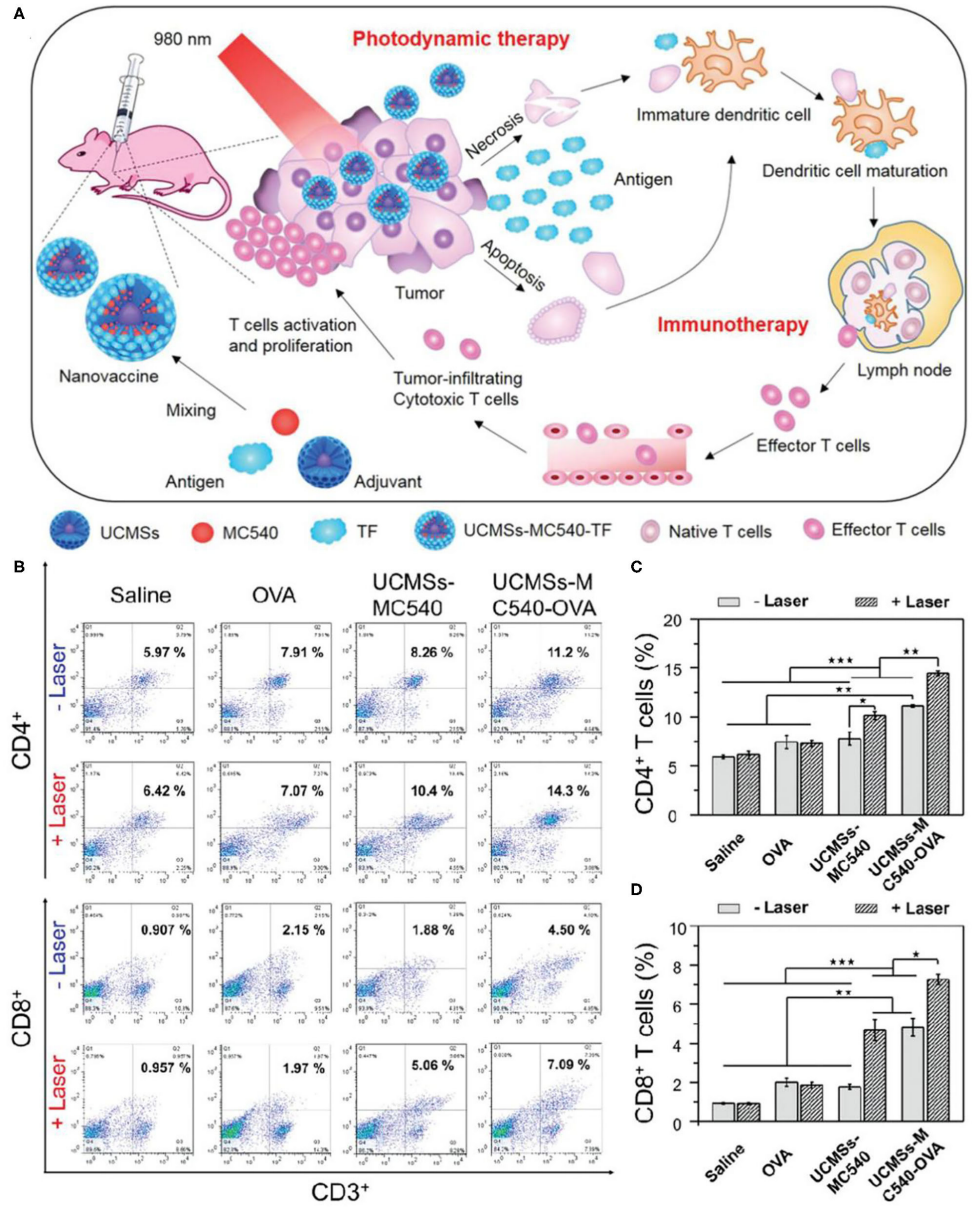
**(A)** Schematic illustration of fabrication and mechanism of UCMSs-MC540-TF nanovaccines for PDT and immunotherapy. **(B)** Flow cytometric analyses of the populations of CD4^+^ (CD3^+^ CD4^+^ as the marker) and CD8^+^ (CD3^+^ CD8^+^ as the marker) T cells in splenocytes of mice immunized by various vaccine formulation. **(C)** The populations of CD4^+^ after different treatments. **(D)** The populations of CD8^+^ after different treatments. Copyright 2018, Wiley-VCH (Ding et al., 2018). *P < 0.5, **P < 0.01, ***P < 0.001.

As mentioned above, phototherapy can induce anti-tumor immune response by inducing apoptosis or necrosis of tumor cells to release TAA. It can be used as *in situ* therapeutic tumor vaccine to activate immune response at the same time of phototherapy. This kind of *in situ* vaccine often needs some help to strengthen the immune response to ensure the effect of immunotherapy. Immune adjuvant can improve the adaptive immune response of the body to the antigen. It can make up for the weak immunogenicity of tumor antigen and enhance the immune response of tumor antigen (Vermaelen, 2019). In order to achieve strong and lasting antitumor effects in cancer immunotherapy, it is very important to select appropriate immune adjuvants (Shao et al., 2015). Mesoporous silica materials have attracted extensive attention from researchers because of their high surface area, adjustable pore size, and good biocompatibility (Lee et al., 2011). Recent studies have shown that mesoporous silica materials can be used as immune adjuvants to enhance the host's antitumor immune effects (Wang et al., 2016a,b). Based on the above studies, Ding et al. prepared monodispersed macroporous mesoporous silica-coated upconversion nanoparticles (UCMSs) with particle size <100 nm and applied them as an immune adjuvant for the first time in antitumor studies (Figure 3A) (Ding et al., 2018). Because of the pore size structure of silica, UCMSs were loaded with PS merocyanine 540 (MC540), chicken OVA or tumor cell fragments simultaneously. Under 980 nm light irradiation, upconverted green light activated MC540 to produce ROS. Furtherly, TAA produced by PDT and released from nanovaccines can stimulate DC maturation, resulting in effector T cell release from lymph node. Finally, T cell activation and proliferation and relevant cytokines release can dramatically kill tumor cells. *In vivo* experiments demonstrated that UCMSs-MC540-OVA exhibited the strongest Th1 and Th2 immune responses and the highest frequencies of CD4^+^, CD8^+^, and effector memory T cells (Figures 3B–D). In addition, UCMSs-MC540-TF could more effectively inhibit the tumor growth and prolong the life span of CT26 tumor-bearing BALB/c mice. The successful construction of this multifunctional UCMSs immune adjuvant opens the way for the development of smart nanomedicines.

**Figure 4:**
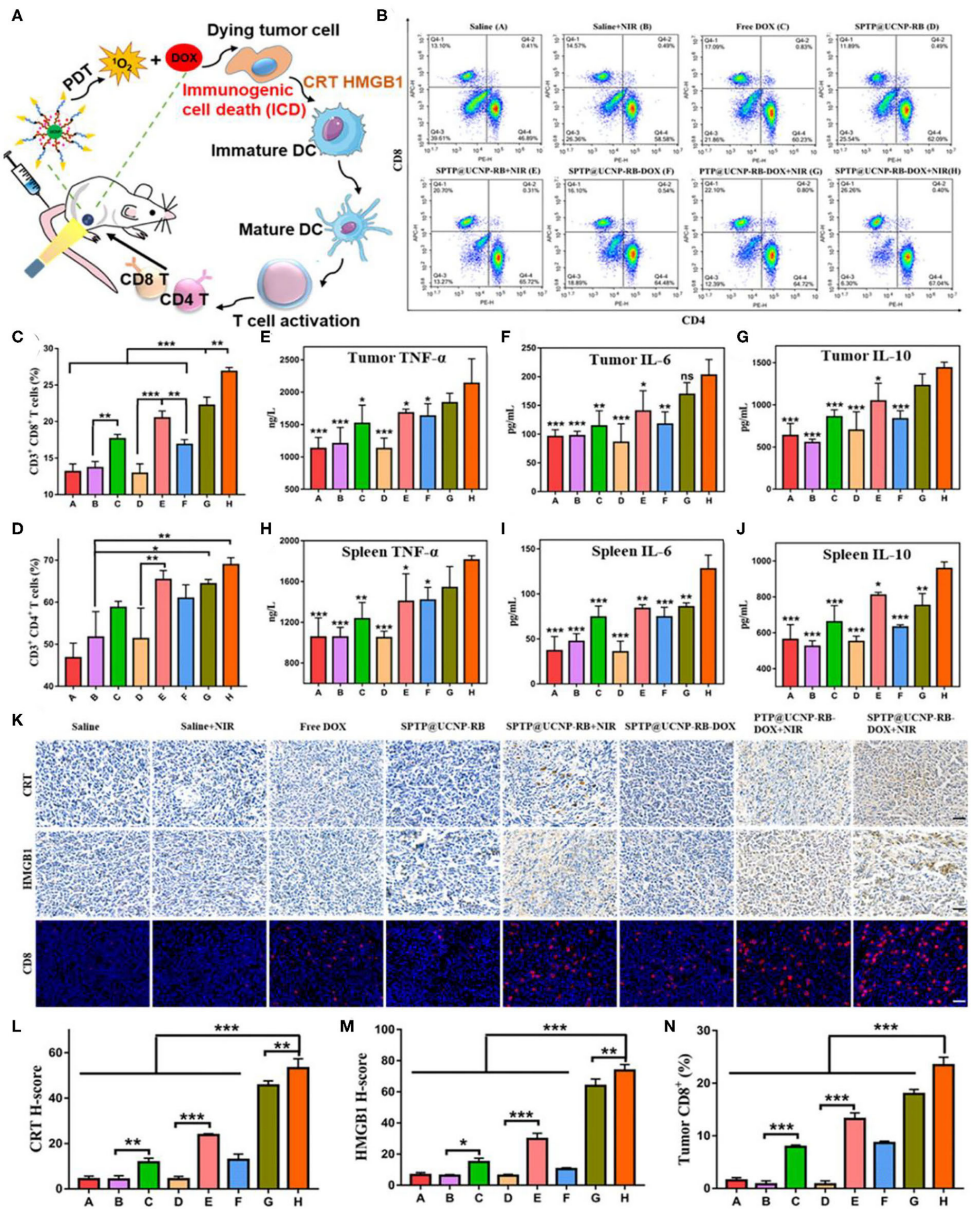
Detection of antitumor immunity *in vivo*. **(A)** Illustration of the ICD-associated DAMP process. **(B–D)** Flow cytometry analysis of CD4^+^ T cells (CD3^+^ CD4^+^) and CD8^+^ T cells (CD3^+^ CD8^+^) proliferation in spleen tissues collected from all mice groups. **(E–G)** Quantitative analysis of TNF-α, IL-6, and IL-10 from tumor lysis solution and **(H–J)** in spleen lysis solution by the Elisa kit. Symbols on columns represent statistic difference vs. the SPTP@UCNP-RB-DOX + NIR group (*n* = 6). **(K)** CRT exposure and HMGB1 secretion in tumor tissues were observed by immunohistochemistry. The infiltration of immune cells in tumors was detected by immunofluorescence dyeing of CD8 T cells (scale bar = 20 μm). **(L–N)** Quantitative analysis of CRT exposure, HMGB1 secretion, and CD8 infiltration in tumor tissues by image J. *p < 0.05; **p < 0.01; ***p < 0.001. Copyright 2020, American Chemical Society (Jin et al., 2020).

Both PDT and drug doxorubicin (DOX) can activate T cell immune responses in TME by activating the ICD process, which make it possible for PDT to be combined with chemotherapy to activate immune response (Garg and Agostinis, 2014; Kuai et al., 2018). The ICD process mainly has adenosine triphosphate (ATP) secretion, heat shock protein (HSP)-antigen peptide complex, calreticulin (CRT) surface exposure, and release of high migration protein B1 (HMGB1) (Garg et al., 2013). Jin et al. loaded chemotherapy DOX and photosensitive rose-bengal (RB) into ROS response micelles based on UCNPs (β-NaYF_4_:Yb,Er), and constructed the treatment nanoplatform (Jin et al., 2020). The ROS response micelles were constructed from biocompatible sialic acid (SA), PEG, thioketone (TK), and poly(lactic-co-glycolic acid) (PLGA). TK is a sulfhydryl-based ROS reactive bond that can trigger rapid drug release through PDT. In addition, SA is a type of N-Acetylneuraminic acid that effectively attaches to E-selectin highly expressed on the cell membrane surface of tumor cells (Xu et al., 2018b), which can dramatically enhance cellular and tumoral uptake of nanoparticles. The micelles (SA-PEG-TK-PLGA, SPTP) loaded with UCNPs, DOX, RB would specifically target overexpressed E-selectin on tumor cells and produced cytotoxic singlet oxygen (^1^O_2_) from RB activated by UCNPs under the irradiation of 980 nm. Furtherly, ^1^O_2_ can destroy the TK linkers and decompose the SPTP micelles system, thus achieving the release of controllable chemotherapy drugs (DOX). The mechanism of the antitumor immunity *in vivo* demonstrated that the synergistic effect of chemo-PDT could induce strong antitumor immune response by the ICD process with enhanced CRT expression, HMGB1 secretion, and sequential activation of T lymphocytes especially CD8^+^ T cells infiltrated in tumors (Figure 4). This process has no addition of immune checkpoint inhibitors or immunoadjuvants, resulting in effective antitumor effects and inhibition of tumor metastasis.

### UCNPs and Tumor Antibody Therapy

**Figure 5:**
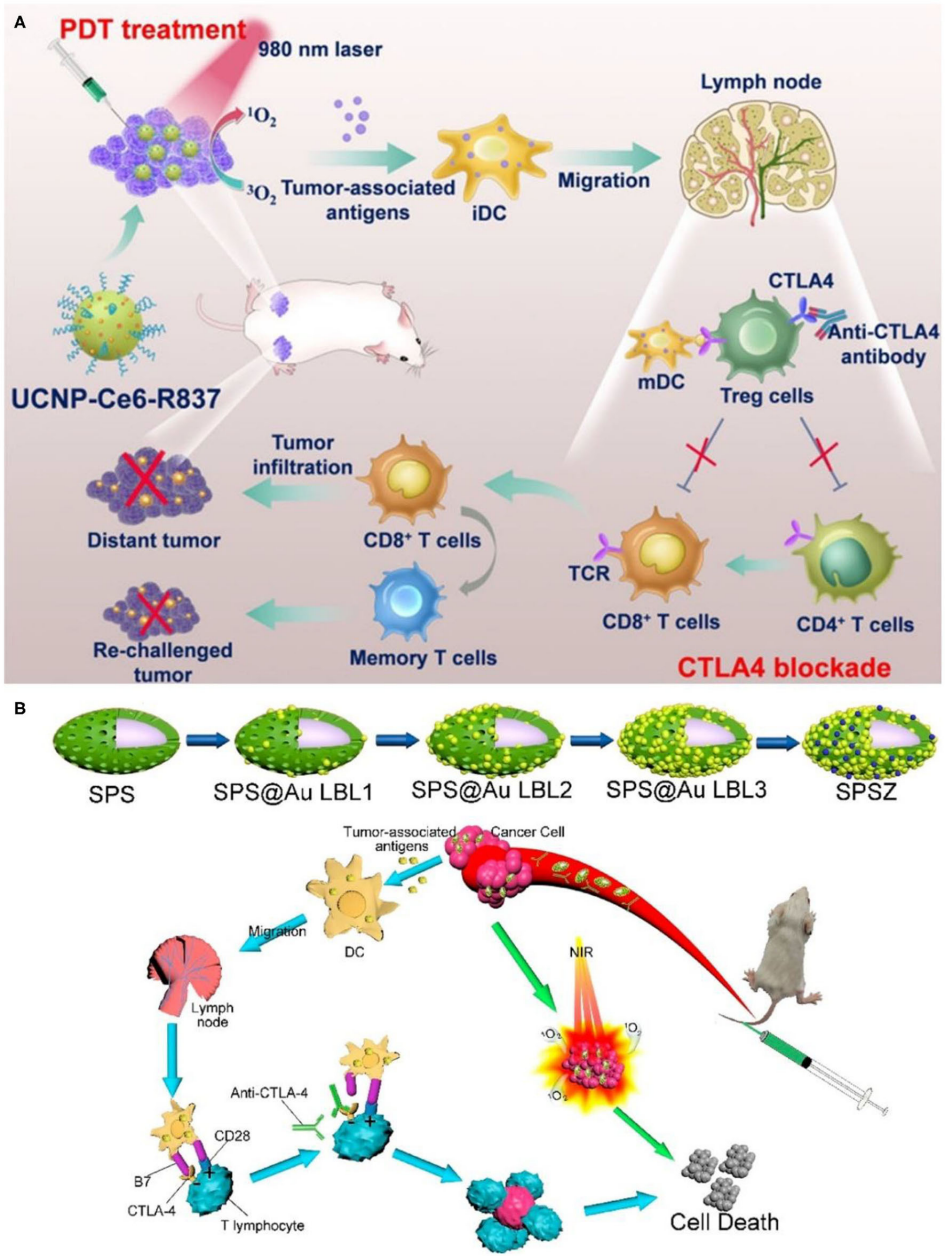
**(A)** Scheme summarizing the mechanisms of combining NIR-mediated PDT with CTLA-4 checkpoint blockade for cancer immunotherapy. UCNP-Ce6-R837 nanoparticles under NIR light would enable effective photodynamic destruction of tumors. The generated TAA in the presence of those nanoparticles as the adjuvant are able to promote strong antitumor immune responses, which with the help of a CTLA-4 checkpoint blockade would eliminate primary tumors under direct NIR exposure, inhibit the growth of distant tumors left behind after PDT, and further yield a long-term immune memory to prevent tumor reoccurrence. Copyright 2017, American Chemical Society (Xu et al., 2017a). **(B)** Schematic diagram of spindle-like of UCNP@SiO_2_@Au synthesis and anticancer therapy. Copyright 2020, American Chemical Society (Lin et al., 2020).

In many cases, the immune effect induced by PDT is not enough to inhibit the growth of residual tumor. The use of immunomodulatory antibodies such as anti-CTLA-4 and anti-PD-1 has high significance in enhancing such immune response. This *in situ* vaccine combined with immunomodulatory antibody can produce immune memory effect and obtain better therapeutic effect. Xu et al. constructed a therapeutic nanoplatform loaded with PS chlorin e6 (Ce6) and immune adjuvant R837 based on UCNPs [NaYF_4_ (Y/Yb/Er = 78:20:2)] (Xu et al., 2017a). As shown in Figure 5A, under 980 nm NIR light irradiation, UCNPs destroyed primary tumors by activating the PS Ce6 to produce cytotoxic singlet oxygen. After PDT treatment, TAA released from tumor residues can effectively promote the maturation of DCs and activate antitumor specific immune responses with the assistance of immune adjuvant R837. After PDT treatment, the levels of immune-related cytokines (IL-12p40, IFN-γ, and TNF-alpha) were significantly increased in UCNPs-Ce6-R837 group. CTLA-4 checkpoint inhibitors can inhibit the immunosuppressive regulatory capacity of regulatory T cells (Wing et al., 2008). UCNPs-Ce6-R837 combined with CTLA-4 immune checkpoint inhibitors not only inhibited the growth of primary tumors, but also had therapeutic effects on distal untreated tumors. It is well-known that immune memory response plays an important role in protecting the body from the second attack of pathogens such as tumor cells (Teixeiro et al., 2009; Kinjyo et al., 2015). The experiment proved that UCNPs-Ce6-R837 combined with CTLA-4 immune checkpoint inhibitor had good long-term immune memory protection.

Besides, UCNPs often face serious problems of low emission efficiency due to the characteristics of low absorption cross-section, inherent structural defects, and prohibition of dipole transition of rare earth ions (Bigall et al., 2012; Bao et al., 2019). UCL can be effectively enhanced by surface plasmon resonance effect by combining gold or silver nanoparticles with UCNPs (Cheng et al., 2017; Sharma et al., 2020). Lin et al. prepared a spindle-like UCNP@SiO_2_@Au mitochondrial imaging probe (Lin et al., 2020). As shown in Figure 5B, the spindle structure made it easier for the probe to enter the cytoplasm. Gold nanoparticles coated on the surface of layer-by-layer enabled the probe to enhance red UCL under NIR excitation. Spindle@SiO_2_@Au had good biocompatibility and could realize real-time dynamic imaging of mitochondria. Under NIR irradiation, UCNP@SiO_2_@AuNPs combined with ZnPc (labeled as SPSZ) showed good UCL imaging ability as well as PDT effect. In addition, SPSZ combined with anti-CTLA-4 effectively inhibited tumor growth through the synergistic effect of PDT and immunotherapy. The resulting immune memory effect protects the body from tumor reinvasion and inhibits tumor recurrence.

**Figure 6:**
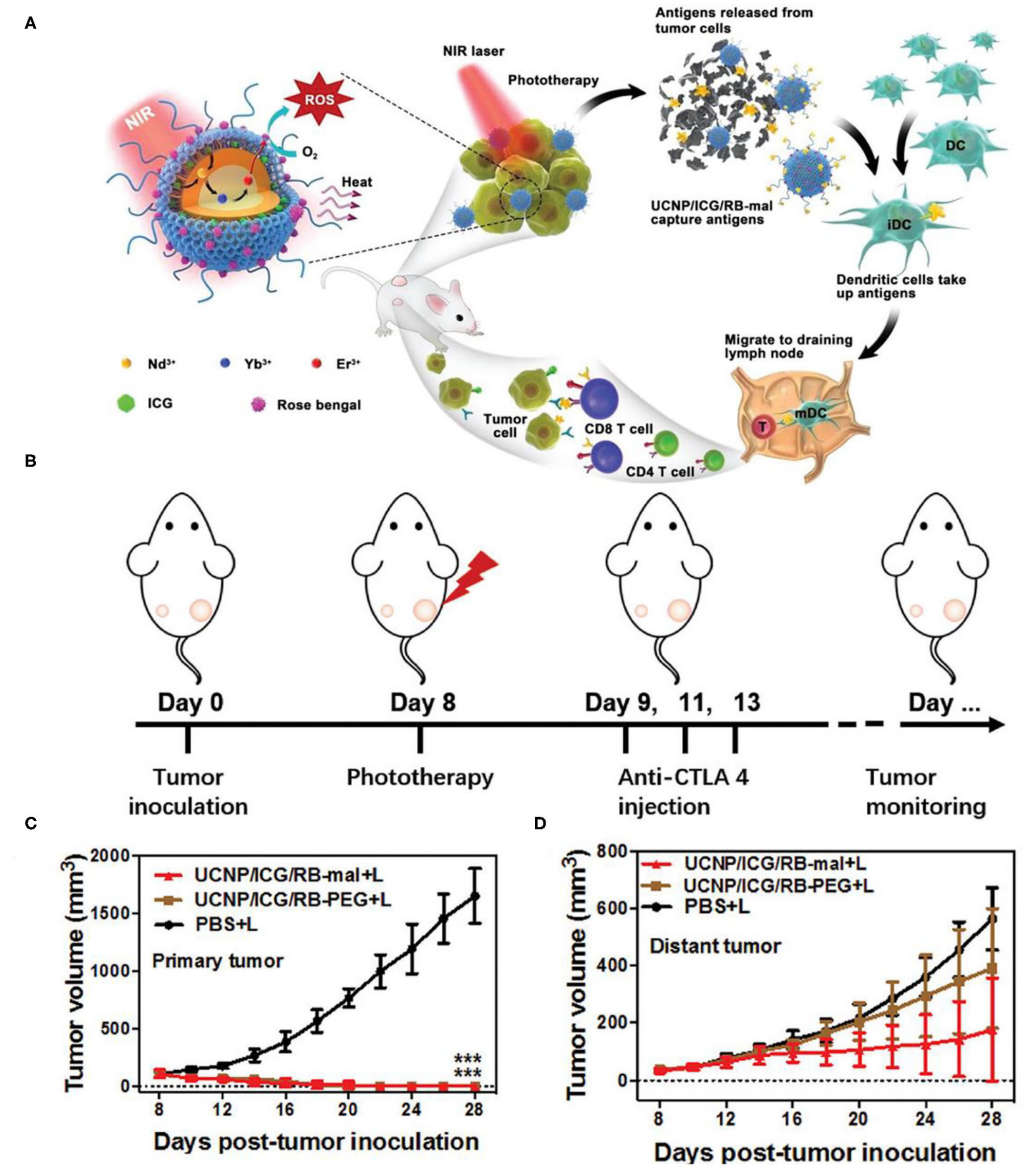
**(A)** Schematic illustration of both fabrication and mechanism of NIR-triggered antigen-capturing nanoplatform for synergistic photo-immunotherapy. Abscopal effect of UCNP/ICG/RB-mal based phototherapy in combination with checkpoint inhibition for simultaneously inoculated tumors. **(B)** Schematic depiction of the experimental approach for the evaluation of the abscopal effect induced by UCNP/ICG/RB-mal based phototherapy. **(C)** Growth curves of primary tumors of mice after various treatments (***P < 0.001 vs. PBS + L group) **(D)** Growth curves of distant tumors on mice in different treated groups. Data are expressed as mean ± SD (*n* = 5). Copyright 2019, Wiley-VCH (Wang et al., 2019a).

Immune checkpoint inhibitors could also cooperate with PDT and PTT to assemble multifunctional therapeutic nanoplatform. *In situ* capture of TAA by nanoplatforms can greatly promote the recognition and uptake of antigens by APCs and enhance the tumor immune response. At the same time, dual modal photothermal and photodynamic agents could be triggered by single wavelength laser to trigger PTT and PDT simultaneously so as to shorten the treatment time (Wang et al., 2013; Yang et al., 2017). Based on this, Wang et al. constructed a multifunctional nanotherapeutic platform using UCNPs (NaYF_4_:Yb,Er@NaYF_4_:Nd) as carriers, loaded with photothermal agents indocyanine green (ICG) and PS RB, and surface-modified lipid molecules (DSPE-PEG-mal) for functionalization (Wang et al., 2019a). Unlike the combination therapy strategy described earlier, the therapeutic nanoplatform UCNP/ICG/RB-mal can capture tumor antigens *in situ* and promote the recognition and uptake of APCs. Due to the large absorption cross-section of ICG, UCNP/ICG/RB-mal produced significant ^1^O_2_ and heat, which could enhance phototherapy and cause more tumor-derived protein antigens (TDPAs) released by ICD (Figures 6A,B). TDPAs captured by UCNP/ICG/RB-mal are taken up by APCs to activate tumor-specific immune responses. Experiments demonstrated that UCNP/ICG/RB-mal+anti-CTLA-4+L treatment significantly inhibited the growth of primary tumors and slowed the growth of distant tumors (Figures 6C,D). UCNP/ICG/RB-mal combined with anti-CTLA-4 synergistically enhanced the antitumor immune response, regulated the tumor immunosuppressive microenvironment, and effectively prevented tumor metastasis. UCNP/ICG/RB-mal multifunctional nanoplatform realized the synergistic effect of PTT, PDT and immunotherapy. This nanoplatform, which selectively destroys primary tumors, increases the immune response rate and eliminates metastatic lesions.

**Figure 7:**
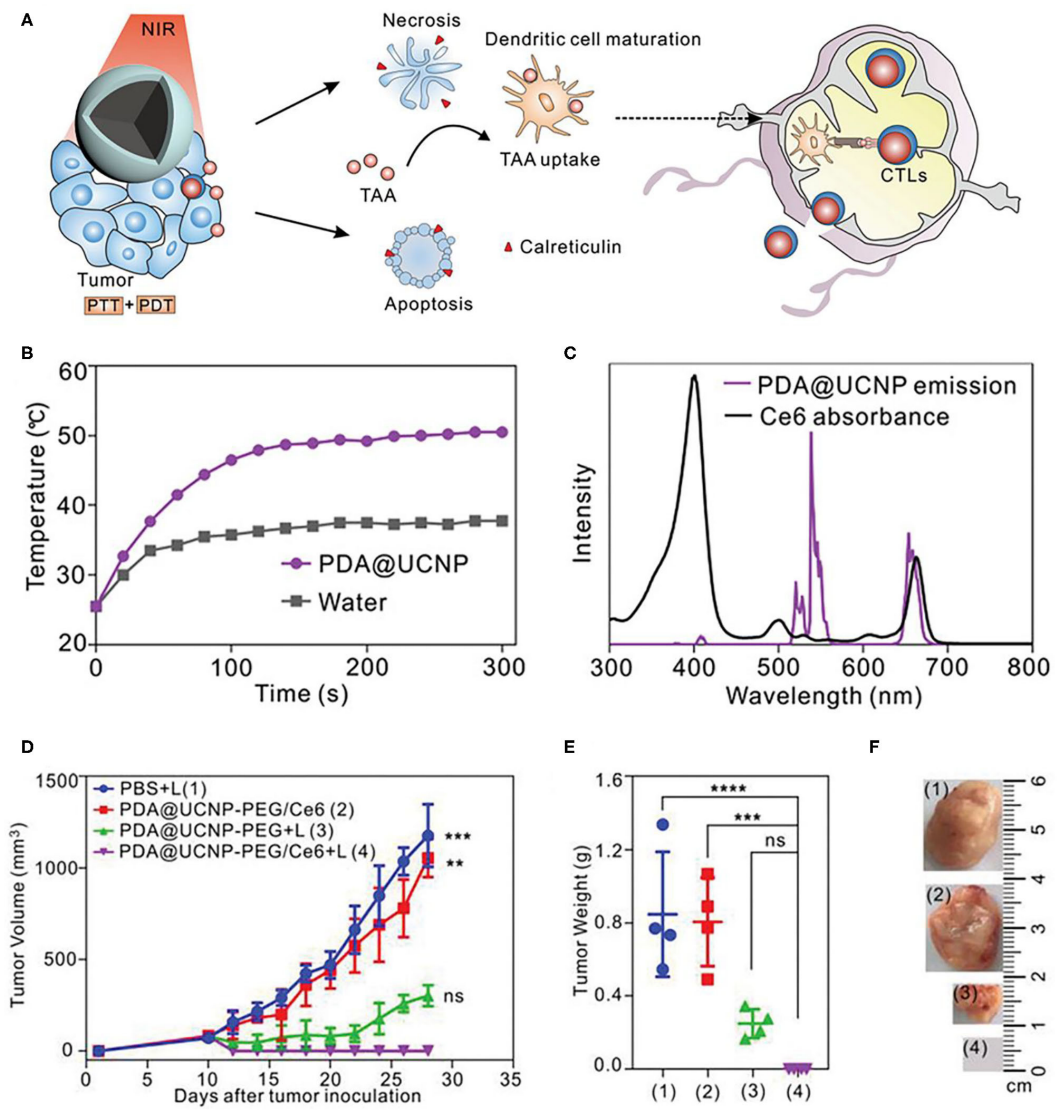
**(A)** Scheme of synergistic phototherapy for augmentation of antitumor immunity. Upon laser irradiation, designed nanoparticles can ablate the primary tumor through synergistic phototherapy, and the released TAA tend to trigger the antitumor immunity which contributes to the inhibition of tumor metastasis and relapse. **(B)** Temperature profiles of pure water and aqueous dispersions of PDA@UCNP nanoparticles (2 mg mL^−1^) as a function of irradiation time (0–5 min). **(C)** The spectrum profiles of Ce6 absorption and PDA@UCNP emission excited with a 980 nm laser. **(D)** Tumor growth curve of 4T1 tumor-bearing mice with various treatments. Weight **(E)** and representative images **(F)** of the tumor from each group after euthanizing the animal on day 28. Copyright 2019, Wiley-VCH (Yan et al., 2019). **P < 0.01, ***P < 0.001, ****P < 0.0001 and ns: not significant (*P* > 0.05).

**Figure 8:**
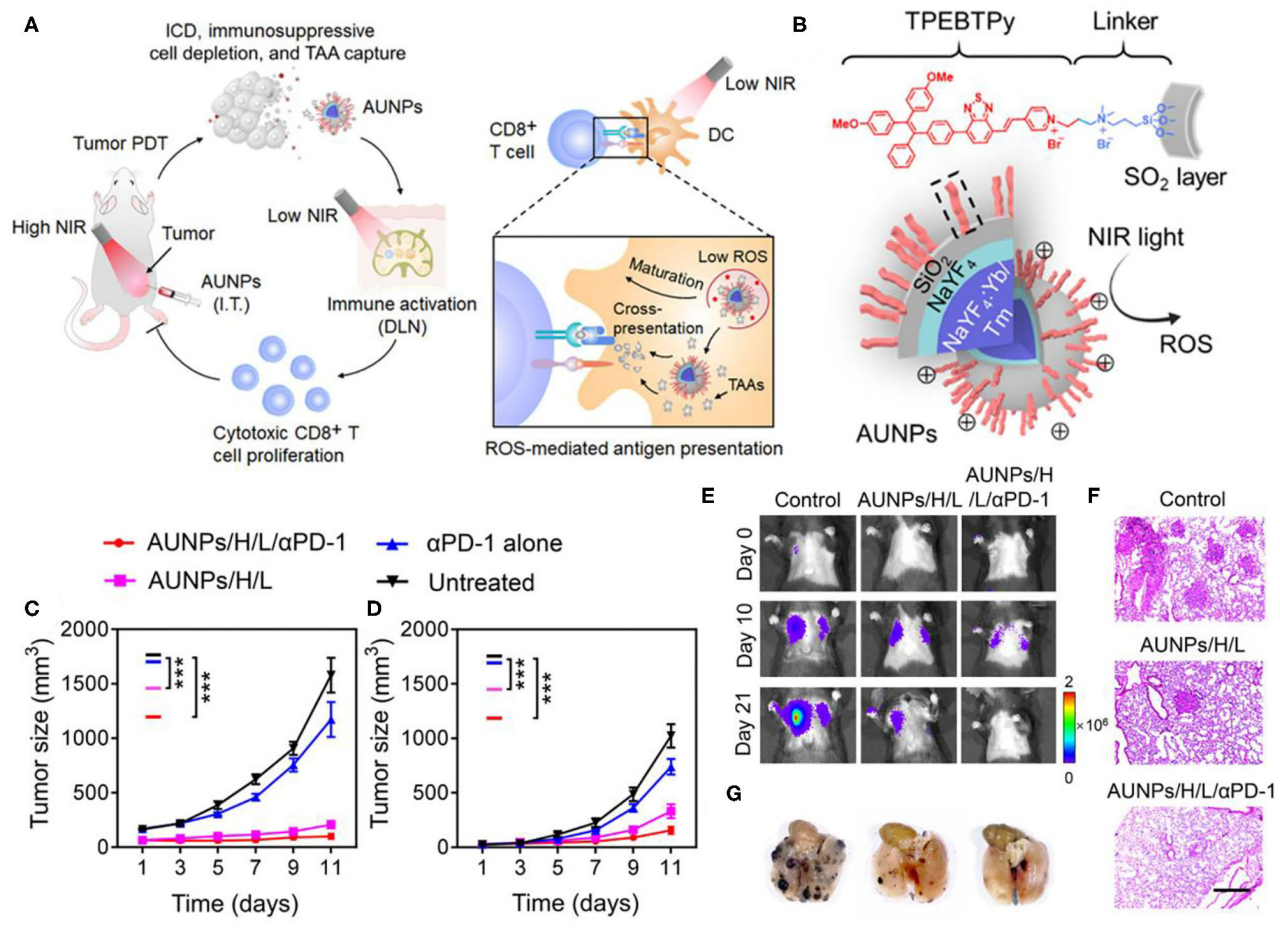
**(A)** Proposed scheme of dual-mode ROS-driven tumor immunotherapy. **(B)** Structure of TPEBTPy and AUNPs. Dashed box indicates a linked TPEBTPy molecule on the AUNPs. **(C)** Primary and **(D)** distant tumor growth curves of the B16F10 tumor–bearing mice with different treatments (*n* = 5). **(E)** Bioluminescence images of mice after rechallenging with intravenous injection of B16F10 tumor cells. **(F)** Representative lung photographs (21 days) and **(G)** hematoxylin and eosin (F&G) images of lung tissue slices from control (naïve mice), AUNPs/H/L, and AUNPs/H/L/αPD-1 groups. Scale bar, 100 μm. Copyright 2020, American Association for the Advancement of Science (Mao et al., 2020). *P < 0.05, **P < 0.01, and ***P < 0.001.

Besides CTLA-4, PD-1 is another crucial inhibitory regulatory receptor that overexpressed on the activated T cells. Anti-PD-1, or anti-PD-L1 are also familiarly used in enhancing the immune effect of ICD derived from phototherapy. Yan et al. prepared polydopamine (PDA) nanoparticles with NaGdF_4_:Yb,Er upconversion shell coated on the surface and then grafted Ce6 molecules to obtain PDA@UCNP-PEG/Ce6 nanoparticles (Yan et al., 2019). As shown in Figure 7A, under single 980 nm NIR light irradiation, PS Ce6 and photothermal agent PDA in nanocomposites were activated to produce excellent PDT and PTT effects (Figures 7B,C), thereby killing tumor cells. Controlled trials showed that the primary tumor was eliminated by PDA@UCNP-PEG/Ce6 with light (Figures 7D–F). In addition, PDT and PTT can induce ICD and activate tumor-specific immune response. PDA@UCNP-PEG/Ce6 combined with immune checkpoint inhibitor α-PD-1 synergistically enhanced the antitumor immune response, not only eliminating primary tumors, but also inhibiting the growth of distal tumors. At the same time, the nanotherapeutics promoted the activation of memory T cells and effectively prevented the recurrence of tumors. The synergistic effect of PDT, PTT and immunotherapy greatly enhances the antitumor effect and is of great significance for the elimination of tumors. In a further work, the same group designed a multifunctional immunostimulant by combining aggregation-induced emission (AIE) PS TPEBTPy with UCNPs to realize the controllable ROS production for PDT and immunotherapy, as shown in Figure 8B (Mao et al., 2020). TPEBTPy with AIE properties showed strong fluorescence and ROS production in the state of aggregation (Qian and Tang, 2017; Hu et al., 2018a). As a NIR antenna, UCNPs can transmit the energy of NIR photons to AIE PS, thus producing a large amount of ROS in deep tissues. In addition, the design of positively charged PS not only facilitated close contact between oxygen and PS, and promoted the rapid diffusion of ROS, but also ensured the subsequent antigen capture through electrostatic interaction. AIE luminogen (AIEgen)–coupled UCNPs (AUNPs) can regulate intracellular ROS levels by controlling NIR irradiation. As shown in Figure 8A, on the one hand, under high power NIR irradiation, a large amount of ROS can effectively kill tumor cells and release TAAs. On the other hand, under low power NIR irradiation, a small amount of ROS activated local DCs and effective cross-presentation, thus inducing the proliferation of stronger CD8^+^ T cells. AUNPs further combined immune checkpoint blockade therapy αPD-1 to improve immune memory, kill the primary tumor cells, and inhibit the growth of the distant tumors (Figures 8C–G). The dual-mode activated ROS strategy provided a reference for the development of tumor immunotherapy.

**Figure 9:**
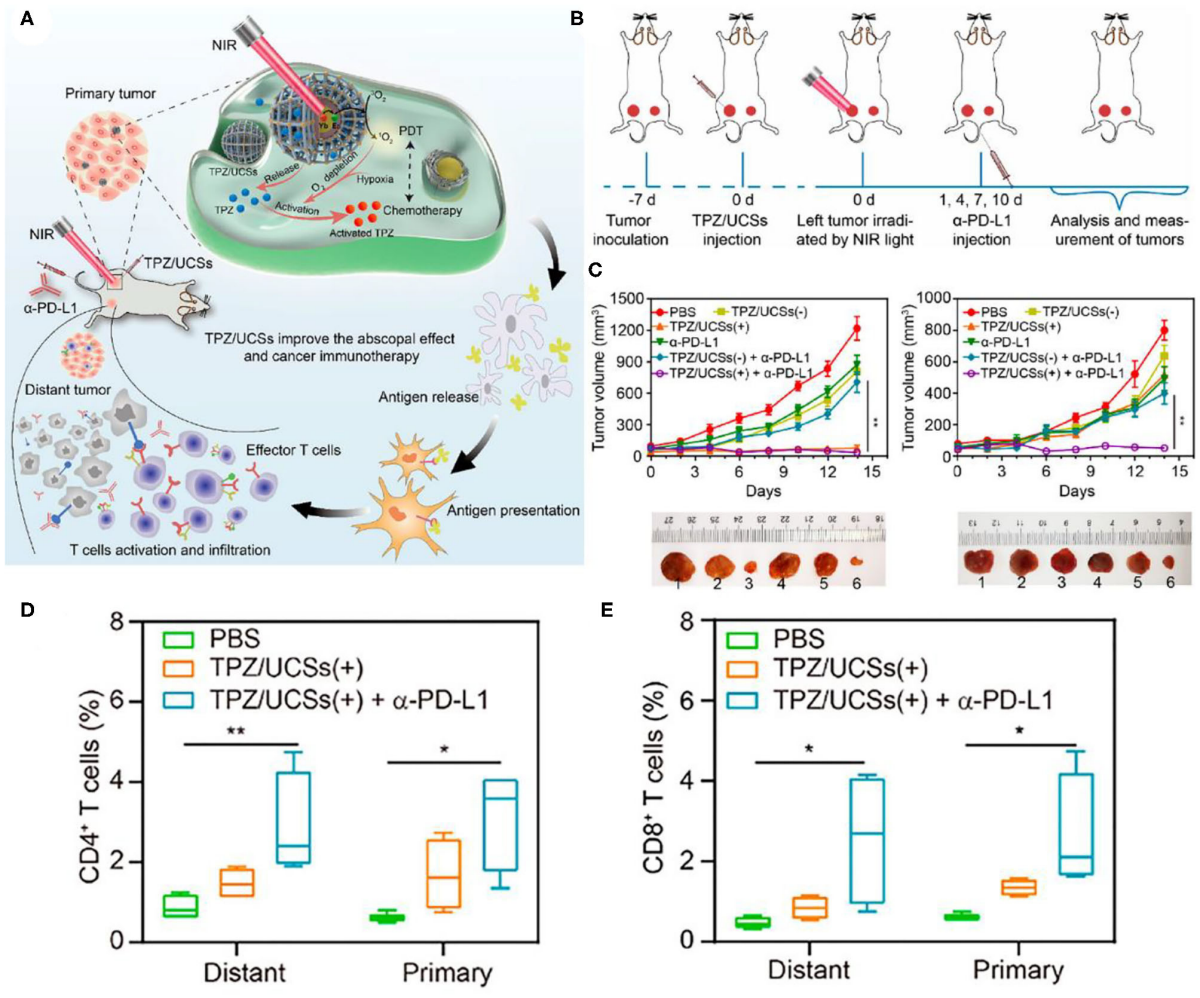
**(A)** Schematic illustration of the structure of TPZ/UCSs and their application to tumor treatment through a combination of NIR light-triggered PDT and hypoxia-activated chemotherapy with immunotherapy. **(B)** Schematic illustration of NIR light-triggered combinational therapy. **(C)** Growth curves of primary tumors (left) and distant tumors (right) in CT26 tumor-bearing mice after different treatments. Photographs of primary tumors and distant tumors collected from mice 14 days after treatments: (1) PBS, (2) TPZ/UCSs(–), (3) TPZ/ UCSs(+), (4) α-PD-L1, (5) TPZ/UCSs(–) + α-PD-L1, and (6) TPZ/UCSs(+) + α-PD-L1. Percentage of tumor-infiltrating **(D)** CD4^+^ T cells, **(E)** CD8^+^ T cells in total tumor cells. Data are means ± SD; *N* = 4. *P < 0.05, **P < 0.01. Copyright 2020, American Chemical Society (Shao et al., 2020).

In some PDT-based systems with porphyrin derivatives and their analogs as PS, the lack of hydrophobicity and tumor selectivity of porphyrin derivatives limits the clinical application of PDT (Cheng et al., 2014). The porphyrin-based metal-organic frameworks (MOFs) have periodic structural ordering, which makes PS molecules in the state of mutual separation, thus avoiding the self-quenching caused by the molecular aggregation of PS (Lu et al., 2014, 2015). In addition, the rich pore structure of MOFs provides a channel for ROS production to spread rapidly, which is conducive to improving the tumor killing effect of PDT (Park et al., 2016). Since the effective energy transfer from UCNP to PS is necessary to achieve NIR photoinduced PDT (Xu et al., 2017b), the attachment of a sufficient number of PSs near UCNP is crucial for the effective generation of ^1^O_2_. Shao et al. designed the core-shell UCNPs@porphyrin MOFs (UCSs) for the combined treatment of hypoxia tumors, as shown in Figure 9A (Shao et al., 2020). High yield synthesis of UCSs was achieved through conditional surface engineering of UCNPs (NaGdF_4_:Yb,Er@NaGdF_4_) and seed-mediated growth strategy. The final structure TPZ/UCSs was obtained by encapsulating the hypoxic activated prodrug tirapazamine (TPZ) in the nanopores of the heterostructure MOF shell. They demonstrated that TPZ/UCSs was a promising system for *in vivo* and *in vitro* cancer treatment through NIR light induced PDT and hypoxic-activated chemotherapy. In addition, the nanoplatform combined with α-PD-L1 immunotherapy promoted complete inhibition of the growth of the distal untreated tumor by producing specific tumor-infiltrating cytotoxic T cells (Figures 9B,C). Compared with the PBS control group, the proportion of CD4^+^ and CD8^+^ T cells was significantly increased after TPZ/UCSs (+) + α-PD-L1 treatment (Figures 9D,E). TPZ/UCSs combined with α-PD-L1 therapy not only inhibited the growth of primary tumors but also slowed the growth of distant tumors.

**Figure 10:**
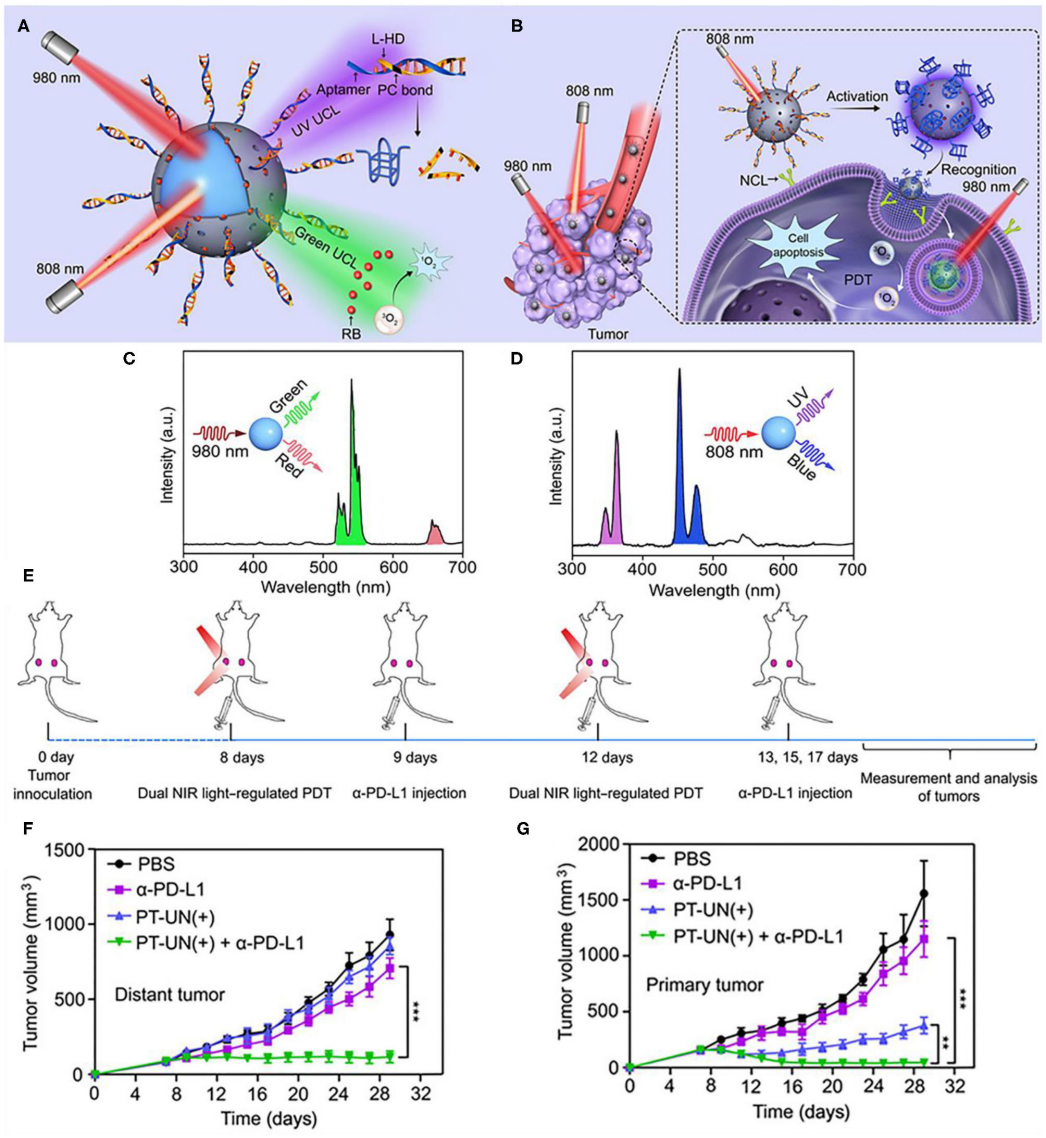
Schematic showing the orthogonal regulation of DNA nanodevice for programmed tumor cell recognition and treatment. **(A)** The orthogonal photoactivation behavior of the DNA nanodevice in response to two NIR light of different wavelengths. **(B)** Sequential activation of the nanodevice with orthogonal UCL for programmed tumor recognition and PDT. UCL spectra of the core-multishell UCNPs upon excitation of 808 **(C)** and 980 nm **(D)**. **(E)** Schematic illustration of combining PT-UN(+) with α-PD-L1 therapy to inhibit tumor growth at both primary and distant sites. Tumor growth curves of primary tumors **(F)** and distant tumors **(G)** of bilateral tumor-bearing mice with different treatments. Data are means ± SD (*n* = 5). Copyright 2020, American Association for the Advancement of Science (Di et al., 2020). **P < 0.01, ***P < 0.001.

Immune checkpoint inhibitors could also corporate with light controlled molecular nanodevices to enhance antitumor efficacy. Di et al. designed an orthogonal upconversion nanoplatform, which achieves NIR light deep tissue penetration and specific target recognition of tumors by DNA nanodevices (Di et al., 2020). Therein, DNA is a highly programmable component that can be used to design molecular nanodevices with specific functions, such as biosensing and imaging (Chen et al., 2015), molecular information calculation (Amir et al., 2014), and controlled drug delivery and release (Brodin et al., 2015; Hu et al., 2019a). The adaptor module L-Apt (UV light–activatable aptamer modules) can be activated by UV light, releasing an adaptor that can recognize the overexpressed recognizes nucleolin on the surface of cancer cells (Reyes-Reyes et al., 2010; Li et al., 2014). Nanodevices (PT-UN) with orthogonal UCL are constructed from PS on the surface of UCNPs (NaGdF_4_:Yb,Er@NaYF_4_@NaYF_4_:Yb,Tm@NaYbF_4_:Nd@NaYF_4_) and L-Apt. With the light regulators UCNPs, UV UCLs obtained under 808 nm irradiation could cleave photocleavage bonds and release aptamers at the required time and guide nanoparticles to target tumor cells. And green UCLs obtained under 980 nm irradiation can stimulate PS to produce cytotoxic ROS, thereby killing tumor cells (Figures 10A,B). Under 808 nm NIR illumination, the characteristic Tm^3+^ emissions located at UV (347 and 363 nm) and blue (452 and 475 nm) regions were observed (Figure 10D). In contrast, visible green (522 and 541 nm) and red (656 nm) emission of Er^3+^ occupied the spectra under NIR excitation at 980 nm (Figure 10C). PT-UN-mediated PDT combined with α-PD-L1 elicited a strong systemic antitumor immune response by promoting the infiltration of effector T cells. The combination of nanodevices with targeted and killing effects and immune checkpoint inhibitors α-PD-L1 not only eliminated the primary tumors, but also inhibited the growth of distal tumors (Figures 10E–G).

**Figure 11:**
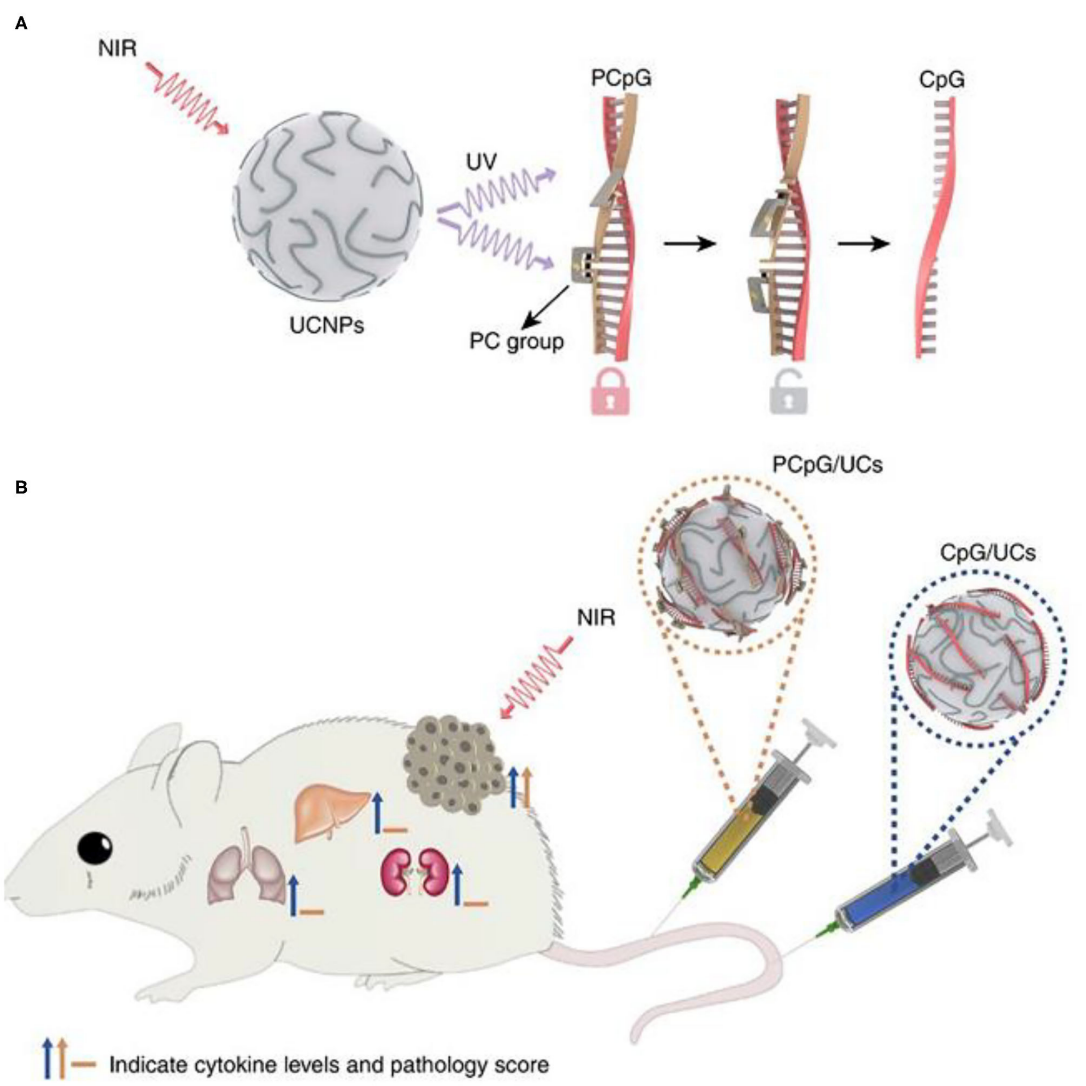
Programming of photoactivatable immunodevice. **(A)** Schematic of the design of photoactivatable immunodevice through the integration of UCNPs with the UV light-responsive PCpG. UCNPs act as transducer to upconvert NIR light into UV light locally, thus liberate CpG ODN from PCpG to achieve the refined temporal control on its immunoactivity. **(B)** Illustration of the photoactivatable immunodevice, PCpG/UCs, for spatially selective triggering of immunoactivity through NIR light irradiation. In contrast to traditional CpG delivery system (CpG/UCs), PCpG/UCs is amenable to personalizing the antitumor modality with reduced systemic toxicity. Copyright 2019, Nature Publishing Group (Chu et al., 2019).

Of course, the application of rare earth nanomaterials in second NIR window (NIR-II, 1,000–1,700 nm) is also involved. Different from the upconversion luminescence of NIR region, NIR-II region is usually down-shifting luminescence, which is used for biological imaging research. In recent years, more and more attention has been paid to the study of molecular probes imaging in the NIR-II window (Hong et al., 2014; Antaris et al., 2017). Imaging at the long wavelength end of the NIR-II window (NIR-IIb, 1,500–1,700 nm) has been reported to increase the penetration depth to subcentimeter and completely eliminate self-fluorescence (Diao et al., 2015a,b; Zhang et al., 2018). At present, clinical *in vitro* immunodiagnosis relies on biopsy to analyze the expression status of tumor cells PD-L1 and the presence/proportion of tumor-infiltrating immune cells. *In vivo* imaging relies on positron emission tomography to solve the problem of uneven distribution of PD-L1 (Mall et al., 2016; Chatterjee et al., 2017). However, no technology had been developed to detect two or more immune-related factors simultaneously *in vivo* with NIRII. Zhong et al. developed the anti-PD-L1 mAb-labeled α-phase Er nanoparticles (ErNPs) with zinc doping (Zhong et al., 2019). Compared with the brightest β-Phase ErNPs before (Zhong et al., 2017), the down-shifting luminescence is enhanced by about 11 times through enhancing the relaxation of multiple phonons in α-phase ErNPs over β-phase, and the symmetry of the crystal field is reduced by doping Zn^2+^. In addition, the designed hydrophilic polymeric cross-linking network endows ErNPs excellent water solubility and biocompatibility. They combined anti-PD-L1 mAb-labeled ErNPs (targeted at PD-L1) and anti-CD8 mAb-labeled PbS quantum dots (targeted at CD8^+^ T cells) for bi-channel molecular imaging to monitor two immune-related molecular targets in real time within the same NIR-IIb emission window (~1,600 nm). Both ErNPs-aPD-L1 excited by 980 nm and PbS-aCD8 excited by 808 nm can emit fluorescence at the NIR-IIb emission window (about 1,600 nm) (Figure 11C). The difference is that the fluorescence lifetime of ErNPs is millisecond and that of PbS quantum dots is microsecond. Two fluorescent signals can be captured by setting the delay in the InGaAs CCD camera. In addition, the PbS-aCD8 channel had higher peripheral signals in the tumor and extends inwards. It was demonstrated that tumors treated with anti-PD-L1 monoclonal antibody produced a strong antitumor immune response and had a high (T/spleen)_CD8 ratio. The assessment of the *in vivo* non-invasive biological distribution of tumor cells and immune cells throughout the body can complement *ex vivo* biopsy-based diagnostic methods.

### UCNPs and Biological Response Modulators

In immunotherapy based on BRMs, systemic injection of immunostimulants is usually required. However, systemic injection of immunostimulants requires precise control of the injection dose, otherwise it will cause serious side effects, such as excessive immune response and excessive release of cytokines (Morgan et al., 2010; Sharma and Allison, 2015). Therefore, it is of great significance to develop a highly spatiotemporal control immune response strategy for tumor site. Chu et al. designed and constructed a NIR light-controlled immunotherapy nanomaterial, which can remotely control the activation of immunotherapy *in vivo* and reduce the systemic toxicity of the nanomaterials (Chu et al., 2019). CpG-ODNs (CpG oligonucleotides, an immunotherapeutic agent) are hybridized with complementary ssDNA (PcDNA) containing photocleavable (PC) bonds to form PCpG, which prevents CpG-ODNs from binding to TLR9. They combined UCNPs (NaGdF_4_:Yb/Tm@NaGdF_4_) with PCpG to construct light activated immune devices. Under the NIR light irradiation, the NIR light of UCNPs was transformed into ultraviolet. As shown in Figure 11A, UV light can photolysis PC bond and release CpG ODNs, thus achieving NIR light controlled immune response. CpG binds to TLR9 in APCs, thus promoting the maturation of DC and the production of inflammatory cytokines, leading to T cell-mediated antitumor immune response (Adamus and Kortylewski, 2018). Compared with the traditional CpG delivery system (CpG/UCS), PCpG/UCS is more suitable for individualized antitumor methods and reduces systemic toxicity (Figure 11B).

In immunotherapy, the use of exogenous chemokines to induce the infiltration of immune cells into tumor can improve the antitumor activity (Homey et al., 2002; Lechner et al., 2011). C-C Motif Chemokine Ligand 21 (CCL21) can stimulate the expansion of CD4^+^ and CD8^+^ T cells and promote the polarization of Th1 cells (Flanagan et al., 2004). In addition, the specific binding of CCL21 to C-C chemokine receptor type 7 (CCR7) expressed on the surface of tumor cells can promote lymph node metastasis of tumor cells (Mashino et al., 2002; Takeuchi et al., 2004). Zhang's group developed a therapeutic nanoplatform to induce the migration of immune cells to tumor lesions and trigger antitumor immune response (Lee et al., 2013). They used UCNPs (NaYF_4_:Yb,Er) as carriers and coated mesoporous silica shell on the surface. Meanwhile, UCNPs@mesoporous silica loads chemokine CCL21. To improve the targeting of UCNPs@mesoporous silica to tumor cells, folic acid (FA) was modified on the surface of the core-shell structure. They developed an *in vitro* endothelial-tumor cell bilayer model and demonstrated that CCL21-FA-UCNPs@mesoporous silica could selectively target folate receptor (FR)-expressing OVCAR-3 cells. In addition, it was confirmed CCL21-FA-UCNPs@mesoporous silica effectively induced T cell migration to tumor cell sites using the Transwell system. On this basis, Zhang's group used microfluidic system to further study the effect of CCL21-FA-UCNPs@mesoporous silica in simulating 3D tumor tissue (Wimalachandra et al., 2019). *In vitro* imaging CCL21-FA-UCNPs@mesoporous silica can be selectively absorbed by FR expressed OVCAR-3 cells. Accumulating in the tumor, CCL21-FA-UCNPs@mesoporous silica induced the migration of CCR7^+^ DCs and Jurkat T cells. This work proved that it is feasible for UCNPs loaded with chemokines to regulate T cell migration.

**Figure 12:**
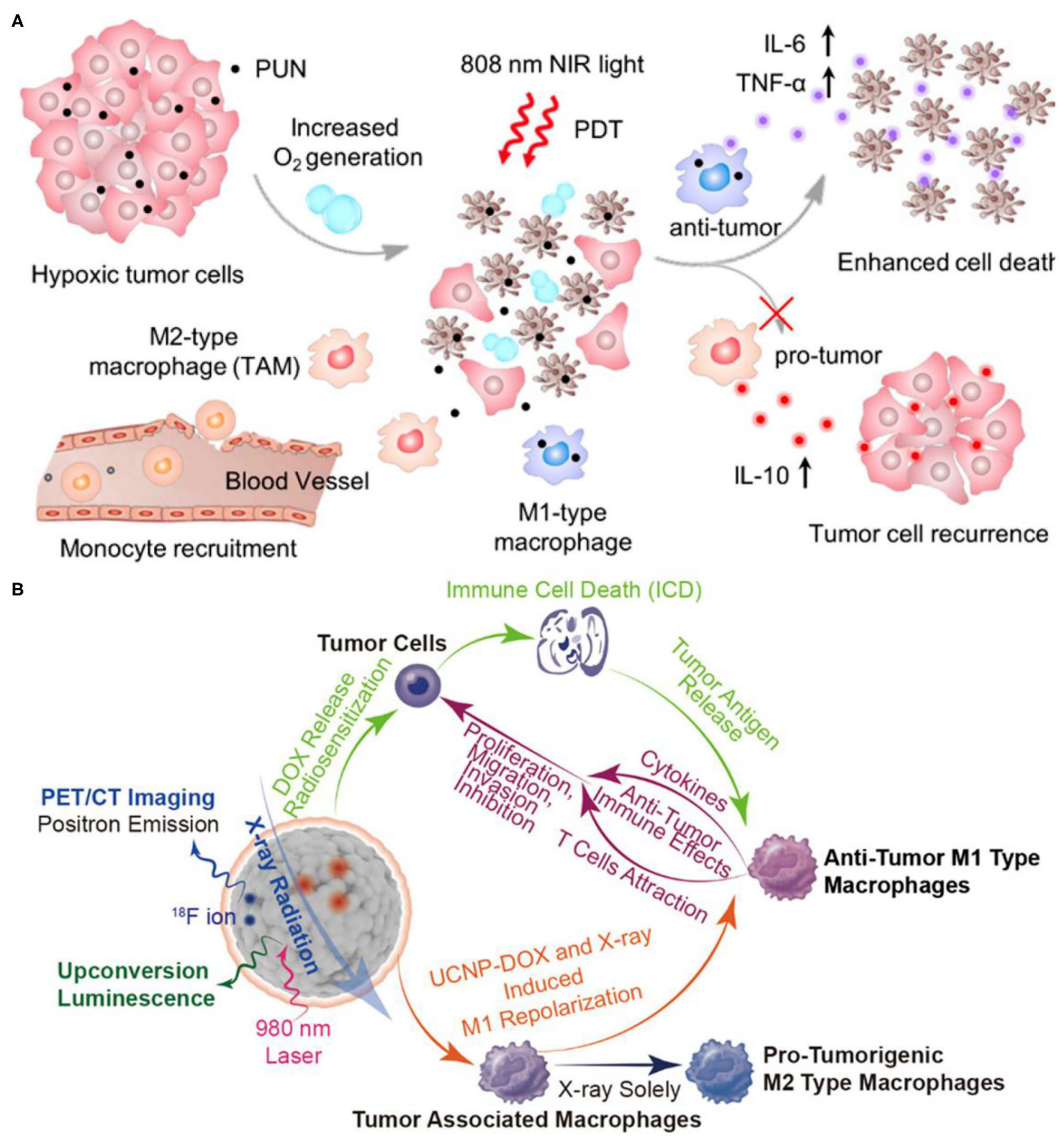
**(A)** Scheme of improved therapy by attenuating hypoxia status and reprogramming tumor-associated macrophages (TAMs) from M2 to M1 phenotype to inhibit the recurrence of tumor cells toward immunotherapy during the post-PDT period. Copyright 2018, American Chemical Society (Ai et al., 2018). **(B)** Mesoporous Bi-Containing radiosensitizer loading with DOX to repolarize tumor-associated macrophages and elicit immunogenic tumor cell death to inhibit tumor progression. Copyright 2020, American Chemical Society (Qin et al., 2020).

In addition to the common BRMs such as cytokines, there are also some chemically synthesized molecules with drug properties, which can also be classified as BRMs because of their potential immunomodulatory functions. Tumor-associated macrophages (TAMs) play an important role in tumor recurrence, invasion, angiogenesis, and metastasis (Quail and Joyce, 2013; Georgoudaki et al., 2016). Tumor-associated macrophages are mainly classified into antitumor and immune-enhancing M1 type macrophages and M2 type macrophages which inhibit immune response, promote angiogenesis, tissue repair, and promote tumor growth (Mosser, 2003). The two phenotypes of TAMs, M1 and M2, will undergo a transition, and their M2 type can be reversely polarized into M1 type TAM, thus triggering the body to produce a specific antitumor immune response, the polarization process is reversible and adjustable (Gordon and Martinez, 2010). The addition of BRMs can affect the polarization process and regulate the immune process. Ai et al. constructed a therapeutic nanoplatform to alleviate tumor hypoxia and synergistically reprogram TAMs population (Ai et al., 2018). They first synthesized UCNPs [NaYF_4_:Yb/Tm/Nd (30/0.5/1%)@NaYF_4_:Nd (20%)], and then prepared UCNs-MnO_2_-Ce6-HA (PUN) nanoparticles using manganese dioxide (MnO_2_), PS Ce6 and hyaluronic acid (HA) for surface functionalization. Under the acidic TME, MnO_2_ can react with endogenous H_2_O_2_ to produce sufficient oxygen, which is conducive to alleviating the hypoxic state of tumors. As shown in Figure 12A, HA can reverse TAM-polarized tumor-promoting M2 phenotype to antitumor M1 phenotype after PDT treatment, which could stimulate tumor-specific immune response and effectively inhibit tumor recurrence, shows the performance of immune regulation (Tran et al., 2015). After PDT treatment, the tumor cells continued to be cultured with PUN, and their cell activity was significantly reduced. This work has reference significance for improving the efficiency of PDT treatment and solving the problem of tumor recurrence after PDT treatment.

On the other hand, TAMs have been reported to make tumors resistant to radiotherapy with a poor prognosis (Barker et al., 2015). After radiotherapy, increased TAMs and secreted growth factors can enhance tumor cell proliferation, invasion, and metastasis (Tsai et al., 2007). The results have shown that nanodrugs can repolarization TAMs and reverse polarize protumor M2-type TAMs into antitumor M1-type TAMs (Rodell et al., 2018). Qin et al. constructed a Bi doped mesoporous upconversion nanophosphor (Na_0.2_Bi_0.8_O_0.35_F_1.91_:20%Yb,2%Er), which were simultaneously loaded with DOX, a chemotherapy drug, and combined with radiotherapy for the treatment of tumors (Figure 12B) (Qin et al., 2020). Co-doping of bismuth ions (Bi ions) in UCNP can enhance UCL, thus Bi-doped UCNP can be used for *in vivo* computed tomography/positron emission tomography/upconversion fluorescence three-mode imaging and radiosensitizer (Lei et al., 2017; Li et al., 2019b; Liu et al., 2019). The radiosensitizer UCNP is combined with chemotherapy drug DOX to enhance the radiation effect, induce tumor ICD, and make protumor M2-type macrophages polarized to antitumor M1-type macrophages. In addition, after UCNP-DOX and X-ray treatment, tumor tissue CD4/CD8 markers showed a large number of CD8 positive T killer cells by immunofluorescence, again confirming the activation of immune effects. The combination of UCNP-DOX and X-ray with three modes of imaging and three treatment methods showed a strong tumor inhibition effect. This work promotes the transformation of radiotherapy from local tumor treatment to systemic treatment, and lays the foundation for the ultimate inhibition of tumor metastasis.

## Conclution and Outlook

Taken together, we summarized the application of UCNPs-based tumor photoimmunotherapy. It has been demonstrated that UNCPs-based PDT can induce ICD, promote dendritic cells maturation, and activate tumor specific immune response. Especially, the combination of UNCPs with various immunity strategies can overcome the limitations of single therapy, which can not only eliminate the primary tumor, but also inhibit the growth of metastatic or recurrent distal tumor.

UCNPs combined with immunotherapy has improved the therapeutic effect of tumor, but it still has challenges and room for growth as detailed below.

First, optimizing energy utilization rate of UCNPs can not only reduce the toxicity from dosage, but also improve the therapeutic effect of nanoparticles. UCNPs are usually combined with PS to produce ROS (He et al., 2017; Shi et al., 2020). However, most PSs can only absorb single emission light by UCNPs with low energy utilization rate. When two PSs excited by different wavelengths were modified to UCNPs, not only the energy utilization rate would be improved, but also more ROS could be produced to enhance the therapeutic effect of tumor. On the other hand, it is also considerable to improve the quantum efficiency of UCNPs by improving the symmetry of crystal field, laser annealing, and doping concentration and surface interface to gain higher energy utilization rate (Tan et al., 2011; Chung et al., 2012).

Second, the most immune checkpoint inhibitors were administered intravenously at a later stage, which act on immune cells universally rather than specific tumor antigens with the risks of inducing side effects (Shao et al., 2020). Linking UCNPs with immune checkpoint inhibitors together in a nanoplatform to release immune checkpoint inhibitors to tumor site through the enhanced permeability and retention (EPR) effect would be a promising strategy to improve the therapeutic efficacy of immunotherapy and reduce immune-related side effects (Li et al., 2019a).

Third, at present, there is no systematic biosafety evaluation on the dispersion, excretion, toxicity, stability, surface loading, and dosage of UCNPs *in vivo* and *in vitro*. In particular, there are few reports on the distribution sites of UCNPs in cells. The potential distribution sites of UCNPs include plasma membrane, lysosome, and cytoplasm, but few in nucleus, endoplasmic reticulum, or mitochondria. On the other hand, the endocytosis of UCNP is complex, involving many factors, such as electrical properties, ligands, culture conditions, and so on (Jin et al., 2011; Zhou et al., 2012). Targeting technology can change the biodistribution sites *in vivo*, especially the cumulative proportion in tumors (Xiong et al., 2009; Yao et al., 2020). The distribution site and UCNPs need further investigation.

Forth, for the distribution of UCNPs *in vivo* after intravenous injection, except for some ultra-small nanoparticles, regardless of the size and surface ligands of UCNPs, the final deposition sites are mainly liver and spleen. The distribution of these organs is related to the injection dose ratio and the size, shape, surface ligands, and time after injection of UCNPs. Recognition and clearance of nanomaterials by cells in the liver, spleen, and reticuloendothelial system may reduce the drug concentration in tumor sites, and shorter blood circulation is also a challenge to be overcome. At present, ligands such as PEG have been widely used to prolong blood circulation (Moghimi et al., 2001). On the other hand, arterial injection, as a method that has been studied for a long time in clinical medicine, provides an opportunity to improve the treatment efficiency of UCNPs by improving UCNPs uptake of tumor.

Fifth, size of nanoparticles has a great influence on its excretion efficiency and toxicity (Cheng et al., 2011). In general, smaller sizes can reduce the *in vivo* elimination time of UCNPs through renal excretion. However, the ultra-small size (such as <5 nm) UCNPs have poor UCL signal due to lattice defects. However, large UCNPs cannot be eliminated by kidney, and the elimination way is mainly through biliary tract, but this way needs more time and has hepatotoxicity. Considering the complexity of UCNPs scavenging process, in addition to the development of UCNPs with both luminous efficiency and scavenging efficiency, more studies on the excretion time of UCNPs may be needed.

Finally, the researches of tumor therapeutic nanoplatforms have sprung up in an endless stream. Most of the current tumor researches are based on mouse model, which still have some differences with human tumor. Some nanodrugs have obvious effect in mouse model, but not in human body. In addition, immunotherapy has shown excellent inhibitory effect on metastatic and recurrent tumors, however, further study is needed about controlling the dosage of immunostimulants. Excessive immune reaction can cause severe damage to the immune system of the patient and even the normal tissues.

The occurrence of tumor is not overnight. And the factors involved are complex and diverse. Similarly, the treatment of cancer cannot be achieved overnight, we need to step out of the dilemma and achieve greater breakthrough. The limitation of single therapy cannot meet our demand for cancer treatment, and the combination therapy is a general trend in the further. How to build a simple and functional treatment nanoplatform needs more enthusiasm and effort of researchers.

## Author Contributions

All authors listed have made a substantial, direct and intellectual contribution to the work, and approved it for publication.

## Conflict of Interest

The authors declare that the research was conducted in the absence of any commercial or financial relationships that could be construed as a potential conflict of interest.
